# Inadequate Folic Acid Intake Among Women Taking Antiepileptic Drugs During Pregnancy in Japan: A Cross-Sectional Study

**DOI:** 10.1038/s41598-019-49782-x

**Published:** 2019-09-18

**Authors:** Yasuko Ikeda-Sakai, Yoshiyuki Saito, Taku Obara, Mikako Goto, Tami Sengoku, Yoshimitsu Takahashi, Hiromi Hamada, Takeo Nakayama, Atsuko Murashima

**Affiliations:** 1 Department of Health Informatics, Graduate School of Medicine and Public Health, Kyoto University, Kyoto, Japan; 2 Department of Pharmaceutical Sciences, Tohoku University Hospital, Sendai, Japan; 3 Environment and Genome Research Center, Graduate School of Medicine, Tohoku University, Sendai, Japan; 4 Tohoku Medical Megabank Organization, Tohoku University, Sendai, Japan; 5 Japan Drug Information Institute in Pregnancy, National Center for Child Health and Development, Tokyo, Japan; 6 Department of Obstetrics and Gynecology, Faculty of Medicine, University of Tsukuba, Tsukuba, Japan; 7 Japan Drug Information Institute in Pregnancy, National Center for Child Health and Development, Tokyo, Japan; 8 Center for Maternal-Fetal, Neonatal and Reproductive Medicine, Department of Perinatology, National Center for Child Health and Development, Tokyo, Japan

**Keywords:** Nutrition, Patient education, Public health, Epidemiology, Neurology

## Abstract

This study aimed to assess characteristics of pregnant women taking antiepileptic drugs with inadequate folic acid intake. This cross-sectional study examined pregnant women taking antiepileptic drugs who were registered in the Japanese Drug Information Institute in Pregnancy (JDIIP) database between October 2005 and December 2016. Participants were classified into two groups according to when they started folic acid supplementation (before pregnancy: ‘adequate’, after pregnancy or never: ‘inadequate’). Logistic regression analysis was performed to investigate factors associated with inadequate folic acid intake. Of 12,794 registrants, 468 pregnant women were taking antiepileptics during the first trimester. Of these, we analysed data from 456 women who had no missing data. As a result, inadequate folic acid intake was noted among 83.3% of them, suggesting that the current level of folic acid intake is insufficient overall. Younger age, smoking, alcohol drinking, multiparity, unplanned pregnancy, and being prescribed AEDs by paediatric or psychiatric departments were independent factors associated with inadequate folic acid intake. As planned pregnancy was the strongest factor, healthcare professionals should ensure that childbearing women taking antiepileptics are informed of the importance of planned pregnancy. In addition, healthcare professionals must gain a better understanding of folic acid intake, as the prevalence of adequate intake differed according to which departments prescribed antiepileptic drugs.

## Introduction

Perinatal use of antiepileptic drugs (AEDs) increases the risk for both congenital malformation and neurodevelopmental impairment in children^1,2^. The risk varies by type of AED and dosage. A comparison of different AEDs revealed that children exposed to valproate sodium had the greatest risk of malformation^1^, with an even higher risk for those whose mothers underwent AED polytherapy as opposed to monotherapy^3^. The presence of physical abnormalities in children of women with epilepsy was more dependent on the use of AEDs during pregnancy than epilepsy itself^4^. Therefore, perinatal AED use should be approached with great caution, as most women with epilepsy must continue AED treatment during pregnancy to ensure adequate seizure control. Notably, AEDs are used to treat a broader range of diseases during pregnancy aside from epilepsy, including headache, neuropathic pain, bipolar disorder, and other mental disorders^5^. Valproate sodium is the only AED with specific instructions to avoid use in childbearing women to the extent possible even in monotherapy in various clinical guidelines^6–8^, and the need to select AEDs with caution is stressed when used in polytherapy^6,7^.

Folic acid supplementation before conception reduces the incidence of neural tube defects in infants^9–11^. Preconceptional folic acid supplementation is recommended for childbearing women, as it is difficult to ensure adequate folate intake solely through diet. In addition, synthetic folic acid has a higher bioavailability than the natural form of folate. In particular, fertile women undergoing AED treatment should take folic acid, because some AEDs promote folate metabolism and lower folic acid blood levels^12^, which may contribute to the impaired cognitive function in children and foetal malformation including neural tube defects. Folic acid supplementation has also been found to reduce the risk of autism in children exposed to AEDs during pregnancy^13^.

Accordingly, childbearing women undergoing AED treatment should ensure that their folic acid intake is adequate. Unfortunately, the current status of folic acid intake among pregnant women taking AEDs is unclear. The present study aimed to clarify folic acid intake among pregnant women taking AEDs and determine characteristics of those for whom intake is inadequate.

## Results

### Setting and participants

**Figure 1:**
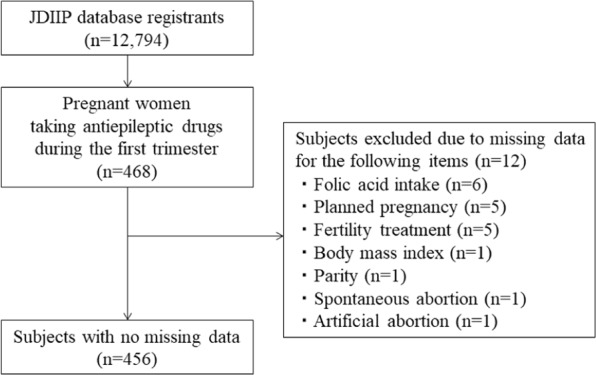
Flow chart for participant screening. JDIIP, Japan Drug Information Institute in Pregnancy.

Data were collected from women registered in the Japan Drug Information Institute in Pregnancy (JDIIP) database. JDIIP provides consultations and collects data on “the influence of medication on pregnant women and nursing mothers/foetuses”. Of the 12,794 registrants in the JDIIP database from October 2005 to December 2016, 468 pregnant women were taking AEDs during the first trimester; we analysed data from 456 of these women who had no missing data. Twelve women were excluded as they were missing data for the following items: folic acid intake (n = 6), planned pregnancy (n = 5), fertility treatment (n = 5), body mass index (n = 1), parity (n = 1), and history of spontaneous and artificial abortion (n = 1) (Fig. 1).

### Participant characteristics

Maternal characteristics are shown in Table 1. Of our study population, 35.1% had planned pregnancies (65.8% among those with adequate folic acid intake, 34.6% among women who started folic acid supplementation after finding out that they were pregnant, and 24.9% among those with no folic acid intake). Mental and behavioural disorders (59.9%) were the most common diseases under treatment, followed by epilepsy (39.0%). AED treatment during the first trimester (Table 2) was undergone as monotherapy in 85.1% and polytherapy in 14.9%. Valproate sodium was the most frequently taken AED (49.3%). The prevalence of valproate sodium use among women with and without planned pregnancies was 37.5% and 55.7%, respectively. Among 225 participants taking valproate sodium, epilepsy, mood disorders, and other diseases with neither epilepsy nor mood disorders were noted as the diseases under treatment by 28.9%, 44.4%, and 35.6%, respectively (Table 3).

**Table 1:** Maternal characteristics.

	Total (n = 456)		Adequate intake		Inadequate intake			
			Before pregnancy (n = 76)		After pregnancy (n = 159)		No intake (n = 221)	
**Age, mean (SD)**	31.0	(5.4)	32.8	(4.8)	30.5	(4.6)	30.7	(5.8)
**Age (category), n (%)**								
< 20 years	9	(2.0)	0	(0)	3	(1.9)	6	(2.7)
21-29 years	165	(36.0)	18	(23.4)	66	(41.3)	81	(36.7)
30-39 years	255	(56.1)	51	(67.5)	83	(52.5)	121	(54.8)
≥ 40 years	27	(5.9)	7	(9.1)	7	(4.4)	13	(5.9)
**Body mass index, n (%)**								
< 18.5	86	(18.9)	15	(19.7)	22	(13.8)	49	(22.2)
≥ 18.5 and < 25	304	(66.7)	53	(69.7)	114	(71.7)	137	(62.0)
≥ 25	66	(14.5)	8	(10.5)	23	(14.5)	35	(15.8)
**Smoking, n (%)**	128	(28.1)	9	(11.8)	37	(23.3)	82	(37.1)
**Alcohol drinking, n (%)**	174	(38.2)	17	(22.4)	67	(42.1)	90	(40.7)
**Parity, n (%)**								
Primiparity	357	(78.3)	67	(88.2)	121	(76.1)	169	(76.5)
Multiparity	99	(21.7)	9	(11.8)	38	(23.9)	52	(23.5)
**Spontaneous abortion, n (%)**	72	(15.8)	18	(23.7)	18	(11.3)	36	(16.3)
**Artificial abortion, n (%)**	100	(21.9)	15	(19.7)	31	(19.5)	54	(24.4)
**Fertility treatment, n (%)**	26	(5.7)	12	(15.8)	12	(7.5)	2	(0.9)
**Planned pregnancy, n (%) Unplanned pregnancy**	160	(35.1)	50	(65.8)	55	(34.6)	55	(24.9)
**Diseases under treatment, n (%)**								
Epilepsy	178	(39.0)	45	(59.2)	73	(45.9)	60	(27.1)
Headache*	18	(3.9)	1	(1.3)	10	(6.3)	7	(3.2)
Other neurological diseases^†^	18	(3.9)	5	(6.6)	5	(3.1)	8	(3.6)
Mental and behavioural disorders	273	(59.9)	35	(46.1)	87	(54.7)	151	(68.3)
Collagen diseases	6	(1.3)	0	(0)	2	(1.3)	4	(1.8)
Others	80	(17.5)	16	(21.1)	22	(13.8)	42	(19.0)

*Headache including migraine and cluster headache.

^†^Other neurological diseases except epilepsy and headache.

**Table 2:** Antiepileptic drug treatment during the first trimester.

	Total (n=456)		Planned pregnancy (n=160)		Unplanned pregnancy (n=296)	
	n	(%)	n	(%)	n	(%)
**Monotherapy or polytherapy**						
Monotherapy	388*	(85.1)	137	(85.6)	251	(84.8)
Polytherapy	68	(14.9)	23	(14.4)	45	(15.2)
**Type of antiepileptic drug**						
Valproate sodium	225	(49.3)	60	(37.5)	165	(55.7)
Lamotrigine	118	(25.9)	54	(33.8)	64	(21.6)
Carbamazepine	80	(17.5)	29	(18.1)	51	(17.2)
Zonisamide	32	(7.0)	13	(8.1)	19	(6.4)
Levetiracetam	26	(5.7)	13	(8.1)	13	(4.4)
Phenobarbital	25	(5.5)	6	(3.8)	19	(6.4)
Phenytoin	14	(3.1)	7	(4.4)	7	(2.4)
Topiramate	11	(2.4)	2	(1.3)	9	(3.0)
Gabapentin	10	(2.2)	2	(1.3)	8	(2.7)
Ethosuximide	1	(0.2)	1	(0.6)	0	(0)
Lacosamide	0	(0)	0	(0)	0	(0)
Perampanel	0	(0)	0	(0)	0	(0)
Primidone	0	(0)	0	(0)	0	(0)

*381 continued the same antiepileptic drug, 7 switched an antiepileptic drug to a different one.

**Table 3:** Diseases under treatment.

	Valproate sodium user (n=225)		Non-valproate sodium user (n=231)	
	n	(%)	n	(%)
**Epilepsy**	65	(28.9)	113	(48.9)
**Headache**	17*	(7.6)	1	(0.4)
**Other neurological diseases**	3^†^	(1.3)	15	(6.5)
**Mental and behavioural disorders**	156	(69.3)	117	(50.7)
Mood disorders	100	(44.4)	84	(36.4)
No mood disorders	56^‡^	(24.9)	33	(14.3)
**Collagen diseases**	0	(0)	6	(2.6)
**Others**	44^§^	(19.6)	36	(15.6)

*1 with epilepsy only, 1 with mood disorders only. 15 with migraine, 2 with cluster headache.

^†^1 with epilepsy only, 2 with brain tumour.

^‡^7 with epilepsy.

^§^5 with epilepsy only, 24 with mood disorders only, 1 with both epilepsy and mood disorders.

### Folic acid intake

**Figure 2:**
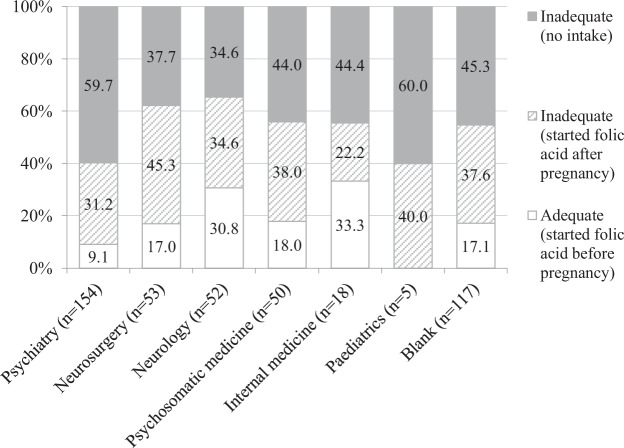
Folic acid intake according to department prescribing antiepileptic drugs. The following departments are not shown. n = 2; Obstetrics and gynaecology. n = 1; Dentistry, Otorhinolaryngology, Rheumatology (all subjects had no folic acid supplementation), Headache clinic, and Anaesthesiology (all subjects had adequate folic acid supplementation).

Folic acid intake included that for both supplements available over-the-counter as well as prescription folic acid. Among all participants, 76 (16.7%) demonstrated adequate folic acid intake before pregnancy, 159 (34.9%) began supplementation after pregnancy, and 221 (48.5%) did not take any (Table 1). Those who received folic acid prescriptions from medical institutions comprised 89 participants (19.5%). Daily doses of folic acid prescription ranged from 0.5 to 20 mg, with 4 mg or more in 70 participants and unclear doses in 5 participants. For supplements available over-the-counter, the exact number of tablets taken daily was recorded only for 3 of 152 participants. Figure 2 shows folic acid intake according to department prescribing AEDs. Most participants were prescribed AEDs by the department of psychiatry (n = 154), followed by neurosurgery (n = 53), neurology (n = 52), and psychosomatic medicine (n = 50). 117 women left blank the department that prescribed AEDs (a “blank” answer meant either “unanswered” or “department other than those listed as options”). The prevalence of adequate folic acid intake ranged from 0 to 33.3% depending on the departments that prescribed AEDs.

### Characteristics of participants with inadequate folic acid intake

Multivariate analysis identified younger age, smoking, alcohol drinking, multiparity, unplanned pregnancy, and being prescribed AEDs by paediatric or psychiatric departments as independent factors associated with inadequate folic acid intake (Table 4). Multicollinearity between explanatory variables was not observed as the maximum of variance inflation factor was 2.18.

**Table 4:** Odds ratio for inadequate folic acid intake by logistic regression analysis.

	Univariable analysis		Multivariable analysis	
	OR	[95%CI]	adjusted OR	[95%CI]
**Age (in increments of 5 years)**	0.68	[0.53-0.86]	0.61	[0.46-0.82]
Body mass index*				
< 18.5	1.00	[0.53-1.88]	0.60	[0.29-1.24]
≥ 25	1.53	[0.69-3.39]	1.99	[0.79-5.01]
**Smoking**	3.39	[1.72-7.51]	2.28	[1.01-5.16]
**Alcohol drinking**	2.44	[1.37-4.35]	2.04	[1.08-3.87]
**Multiparity**	2.31	[1.11-4.82]	3.53	[1.52-8.23]
**Fertility treatment**	0.20	[0.09-0.46]	0.71	[0.26-1.90]
**Spontaneous abortion**	0.53	[0.29-0.97]	0.77	[0.36-1.62]
**Artificial abortion**	1.17	[0.63-2.17]	0.83	[0.40-1.72]
**Planned pregnancy**	0.21	[0.13-0.36]	0.28	[0.16-0.52]
**Valproate sodium use during the first trimester**	1.98	[1.18-3.29]	1.09	[0.60-1.97]
**Polytherapy with antiepileptic drugs**	0.73	[0.38-1.40]	0.50	[0.23-1.06]
**Departments that prescribed antiepileptic drugs** ^†^				
Paediatrics or psychiatry	3.15	[1.65-6.03]	3.04	[1.48-6.24]
Blank^‡^	1.48	[0.82-2.67]	1.82	[0.92-3.59]

*The reference standard is ≥18.5 and <25.

^†^The reference standard is department that is neither paediatrics nor psychiatry, including neurosurgery, neurology, psychosomatic medicine, internal medicine, obstetrics and gynaecology, dentistry, otorhinolaryngology, rheumatology, headache clinic, and anaesthesiology.

^‡^“Blank” regarding the department prescribing antiepileptic drugs meant either “unanswered” or “department other than those listed as options.” Abbreviations:

OR, odds ratio; CI, confidence interval; VPA, valproate sodium.

## Discussion

The present study examined folic acid intake and determined characteristics of those with inadequate folic acid intake among pregnant women taking AEDs, using data from the JDIIP database. Of our study population, 83.3% did not take folic acid before pregnancy. These women with inadequate folic acid intake were more likely to be younger, have a history of smoking or alcohol drinking, multiparous, prescribed AEDs by paediatric or psychiatric departments, and less likely to have a planned pregnancy. The prevalence of adequate folic acid intake ranged from 0 to 33.3% in the various departments that had prescribed AEDs. Mental and behavioural disorders were the most common diseases for which AEDs were prescribed as treatment, followed by epilepsy.

Folic acid intake, including both supplemental over-the-counter and prescription folic acid, was insufficient among pregnant women taking AEDs. Although the prevalence of inadequate folic acid intake in the present study (83.3%) was lower than that of general women in a nationwide population-based cohort study in Japan (92.6%)^14^, the current state of folic acid intake should be improved given that children of pregnant women taking AEDs are at greater risk of developing congenital malformations than those of general childbearing women. Participants who started folic acid intake after pregnancy (comprising one-third of all participants) intended to take folic acid as well as those with adequate folic acid intake. Nonetheless, these participants did not start folic acid intake before finding out that they were pregnant and thus the full effect of folic acid supplementation cannot be expected in such cases. One reason for this may be related to whether or not the pregnancy was planned (planned pregnancies were noted for 65.8% with adequate folic acid intake and 34.6% of those starting folic acid after pregnancy). Further investigation is required to identify factors to explain why some women did not take any folic acid.

Women with planned pregnancies were more likely to have adequate folic acid intake. This was consistent with results from previous population-based studies^15–17^ and a hospital-based study^18^ of general pregnant women. In the present study, a planned pregnancy was the strongest factor associated with folic acid intake. Another study found a non-significant association between planned pregnancy and prescription of folic acid, but women with epilepsy who had planned pregnancies tended to have lower foetal exposure to AEDs through the selection of monotherapy and by avoiding valproate sodium use^19^. The present study also found a lower prevalence of valproate sodium use in those with planned pregnancies than in those with unplanned pregnancies (37.5% versus 55.7%, respectively), even though the prevalence of AED polytherapy was comparable in both of these groups, at approximately 15%. Having a planned pregnancy may improve the mother’s behaviour, such as preconceptional folic acid intake and AED adjustment to allow for lower teratogenic risk. On the other hand, since unplanned pregnancies currently represent the majority, a system may need to be developed to guide women toward adequate folic acid intake prior to attempting to get pregnant. One potential solution would be to implement mandatory national folic acid fortification, as is done in many other countries^20^, but not currently in Japan.

The prevalence of adequate folic acid intake differed by the department that prescribed AEDs. Those who were prescribed AEDs by paediatric or psychiatric departments were less likely to have adequate folic acid intake, potentially due to differences in attitudes among healthcare professionals about the benefits of preconceptional folic acid intake or in preparation for unexpected pregnancies. Although medical treatment policies are not solely dependent on guidelines, actual clinical practice in each department might differ depending on the contents of guidelines and recommendations. In Japan, preconceptional folic acid intake for childbearing women requiring AED treatment is recommended in guidelines for epilepsy^6^ and chronic headaches^7^, but is not mentioned in guidelines for neuropathic pain^8^, bipolar disorder^21^, or schizophrenia^22^. An increased awareness about folic acid is needed among healthcare professionals, as well as among women of childbearing age, and more information must be provided about folic acid supplementation in disease guidelines. Targeted healthcare professionals would include physicians prescribing AEDs as well as those in various other occupations, e.g., pharmacists obligated to refer to doctors when there is any doubt about prescriptions, nurses assisting in medical treatment, and public health nurses performing educational activities. Notably, interpretation of our findings about folic acid intake according to department prescribing AEDs should be made with caution, since approximately 25% of participants left blank the answer regarding the department that prescribed AEDs.

Other characteristics of inadequate folic acid intake identified in this study included younger age, multiparity, and a history of smoking and/or alcohol drinking. These characteristics were consistent with those identified by other studies of general pregnant women^14–17^. Younger women were less likely to have planned pregnancies^17^ and knowledge of folic acid and its benefits^23^, demonstrating the necessity of school health education on this subject as well as continuous efforts to increase the awareness of the importance of folic acid intake.

One questionnaire study revealed that pregnant women obtained information on folic acid through mass media (47%), the Internet (17%), healthcare providers (13%), and the maternal and child health handbook (11%)^24^. The maternal and child health handbook is a booklet given to pregnant women in Japan, contains information on safe pregnancy, childbirth, and children’s health, and is used to record their own health conditions as well as growth parameters of their children. Given the popularity of the Internet and mass media to obtain this information, it may be most effective to use these resources to increase awareness about folic acid intake among pregnant women and those considering pregnancy. Typically, communication with maternity healthcare providers begins after the pregnancy is confirmed, or if pregnancy issues such as spontaneous abortion occur. In addition, women receive their maternal and child health handbooks only after a healthcare professional confirms their pregnancy. Therefore, the impact of promoting preconceptional folic acid intake by either healthcare professionals or through information in the maternal and child health handbook might be limited.

Our study also found inappropriate use of valproate sodium among pregnant women taking AEDs. Perinatal valproate sodium use has been reported to increase the risk of congenital malformations^1^ and decrease IQ scores in children^25^. Indications for valproate sodium use during pregnancy are limited to those with epilepsy or bipolar disorder (a type of mood disorder), and the drug should only be prescribed if all other medications are ineffective in treating the condition or if there are no other alternatives. However, the present study found that 35.6% of pregnant women taking valproate sodium reported diseases other than epilepsy or mood disorders as diseases under treatment. Use of valproate sodium to prevent migraines is contraindicated for pregnant women because the risks outweigh any benefits. On a related note, all non-pregnant women of childbearing age taking valproate sodium to prevent migraines should use effective birth control^26^. Patients with chronic pain disorders or mental disorders other than mood disorders should also be treated in accordance with this policy.

The present study had some limitations worth noting. First, our participants may not be representative of all pregnant women using AEDs, as they had taken some AEDs during pregnancy and voluntarily consulted the JDIIP. That said, the proportion of AED users among pregnant women is very low (0.2 to 2.0%)^5,27,28^, and the present study included more than 450 participants, providing highly valuable data. Second, for supplements available over-the-counter, the exact number of tablets taken daily was recorded only by 3 participants because supplements were not considered medication. However, we estimated their daily doses of folic acid to be approximately 0.4 mg based on records of the product names. Third, since the term “before/after pregnancy” as it applies to when folic acid supplementation began was not strictly defined in the self-report questionnaires, potential misclassification might lead to overesting the prevalence of adequate folic acid intake. The accuracy of information could be improved with further efforts to reduce misclassification and missing data. Fourth, we did not consider the effects of marital status, educational history, or income, all of which are important factors related to folic acid intake. Despite these limitations, we gained valuable insight on pregnant women taking AEDs to treat diseases other than epilepsy because we did not collect data from registries limited to patients with epilepsy.

In conclusion, folic acid intake is currently insufficient among pregnant women taking AEDs. Younger age, smoking, alcohol drinking, multiparity, unplanned pregnancy, and being prescribed AEDs by paediatric or psychiatric departments were independent factors associated with inadequate folic acid intake. Having a planned pregnancy was the strongest factor associated with adequate folic acid intake, and thus healthcare professionals should educate women of childbearing age taking AEDs about the importance of planned pregnancy. Awareness among healthcare professionals including clinicians and pharmacists, among others, should also be increased, as the prevalence of adequate folic acid intake differed depending on which department prescribed AEDs.

## Methods

### Study design and setting

This is a cross-sectional study of data from the JDIIP database. The JDIIP, a project approved by the Ministry of Health, Labour and Welfare, was conducted by the National Center for Child Health and Development beginning in October 2005.

### Participants

Pregnant women who used AEDs during the first trimester of pregnancy and who were registered in the JDIIP database from October 2005 to December 2016 were eligible to participate. Those with missing data for items about maternal characteristics and folic acid intake were excluded. AEDs used by participants included carbamazepine, ethosuximide, gabapentin, lacosamide, lamotrigine, levetiracetam, perampanel, phenytoin, phenobarbital, primidone, topiramate, valproate sodium, and zonisamide. Oxcarbazepine was not included since it has not been released in Japan.

### Data collection

Women desiring a consultation completed a questionnaire form containing information on their medication use and mailed it to apply for consultation. Consultations could occur by three ways: (1) By telephone: a physician/pharmacist responds to common consultations about items such as cold medicine and analgesics; (2) A woman visits one of the 39 JDIIP outpatient clinics nationwide and a medical specialist responds to the consultation; or (3) A woman visits her attending physician, who then provides an explanation based on the response from the JDIIP.

Items in the database were maternal age, height, body weight before pregnancy, smoking status, alcohol drinking, parity, medical history, fertility treatment, planned pregnancy, medication use (product name, generic name, date of start and discontinuation, daily dose, department that prescribed the medication), and folic acid intake (both prescription and supplementary over-the-counter; for the latter, product name and timing of initiation), as collected from the questionnaire surveys. In addition, some women agreed to return a postcard survey on neonatal outcomes to indicate outcomes including congenital malformation. Women who reportedly never smoked or who quit smoking before pregnancy (i.e., before the day the last menstrual period started) were defined as non-smokers and women who reported that they were still smoking or had smoked until pregnancy confirmation were defined as smokers. The same guidelines were used to define alcohol drinking. The definition of a planned pregnancy was that the woman intended her pregnancy. If there was any ambiguity in the description of the questionnaire, research coordinators either confirmed by phone, or pharmacists or doctors provided confirmation at the consultation.

### Outcome and statistical analysis

We assessed maternal characteristics and AED treatment during the first trimester (from 0 weeks 0 days until 13 weeks 6 days of gestation). As maternal characteristics, we extracted information on age, body mass index, smoking status, alcohol drinking, parity, history of spontaneous and artificial abortion, fertility treatment, planned pregnancy, and diseases under treatment. With regard to AED treatment during the first trimester, we extracted information on type of AEDs (e.g., valproate sodium), date of AED use, monotherapy or polytherapy, and the department that prescribed AEDs.

The outcome was prevalence of folic acid intake. We classified participants into two groups, depending on when folic acid intake began: those who began folic acid supplementation before pregnancy were defined as ‘adequate’ intake, while those who started after pregnancy or who never started (no intake) were defined to have ‘inadequate’ intake. Univariable and multivariable logistic regression analyses were performed to calculate the odds ratio and the 95% confidence interval of the association between maternal characteristics as explanatory variables and inadequate intake of folic acid as the outcome variable. Multicollinearity among explanatory variables was confirmed by variance inflation factors. All statistical analyses were conducted with JMP Pro 13.0 (SAS Institute Inc., Cary, NC, USA).

### Ethical considerations

Researchers and other personnel could not identify individuals from analytical data, as the information was input by a research coordinator based on questionnaire forms and postcard surveys for neonatal outcomes. Data were anonymised by an information administrator in the JDIIP. Written informed consent regarding the construction of the JDIIP database was obtained from all subjects. For the purpose of the present study, the opt-out was adopted (we disclosed the information on this study and provided potential participants with the opportunity to refuse to participate). All procedures were conducted in accordance with ethical guidelines for medical and health research involving human subjects established by the Ministry of Health, Labour and Welfare in Japan. All study protocols were approved by the ethics committees of the National Center for Child Health and Development (No. 1502) and Kyoto University (No. R1244-1).

## Data Availability

Datasets generated during the current study are not publicly available due to a lack of agreement from participants and lack of approval from the ethics committees regarding data provision to third parties. However, the datasets are available from the corresponding author on reasonable request for collaborative research and with permission from the National Center for Child Health and Development.
