# Publisher Correction: Inadequate Folic Acid Intake Among Women Taking Antiepileptic Drugs During Pregnancy in Japan: A Cross-Sectional Study

**DOI:** 10.1038/s41598-020-63105-5

**Published:** 2020-04-14

**Authors:** Yasuko Ikeda-Sakai, Yoshiyuki Saito, Taku Obara, Mikako Goto, Tami Sengoku, Yoshimitsu Takahashi, Hiromi Hamada, Takeo Nakayama, Atsuko Murashima

**Affiliations:** 1 Department of Health Informatics, Graduate School of Medicine and Public Health, Kyoto University, Kyoto, Japan; 2 Department of Pharmaceutical Sciences, Tohoku University Hospital, Sendai, Japan; 3 Environment and Genome Research Center, Graduate School of Medicine, Tohoku University, Sendai, Japan; 4 Tohoku Medical Megabank Organization, Tohoku University, Sendai, Japan; 5 Japan Drug Information Institute in Pregnancy, National Center for Child Health and Development, Tokyo, Japan; 6 Department of Obstetrics and Gynecology, Faculty of Medicine, University of Tsukuba, Tsukuba, Japan; 7 Center for Maternal-Fetal, Neonatal and Reproductive Medicine, Department of Perinatology, National Center for Child Health and Development, Tokyo, Japan

Correction to: *Scientific Reports* 10.1038/s41598-019-49782-x, published online 18 September 2019

The original version of this Article contained errors.

Affiliation 7 was a duplicate of Affiliation 5, and it has now been removed. The original Affiliation 8 is now listed as Affiliation 7.

In addition, in the Abstract,

“As planned pregnancy was the strongest factor, healthcare professionals should ensure that childbearing women taking antiepileptics are informed of the importance of planned pregnancy.”

now reads:

“As unplanned pregnancy was the strongest factor, healthcare professionals should ensure that childbearing women taking antiepileptics are informed of the importance of planned pregnancy.”

Moreover, in Table 1 row heading “Planned pregnancy, n (%) Unplanned pregnancy” was incorrect.

**Table 1:** Maternal characteristics.

	Total (n = 456)		Adequate intake		Inadequate intake			
			Before pregnancy (n = 76)		After pregnancy (n = 159)		No intake (n = 221)	
**Age, mean (SD)**	31.0	(5.4)	32.8	(4.8)	30.5	(4.6)	30.7	(5.8)
**Age (category), n (%)**								
<20 years	9	(2.0)	0	(0)	3	(1.9)	6	(2.7)
21–29 years	165	(36.0)	18	(23.4)	66	(41.3)	81	(36.7)
30–39 years	255	(56.1)	51	(67.5)	83	(52.5)	121	(54.8)
≥40 years	27	(5.9)	7	(9.1)	7	(4.4)	13	(5.9)
**Body mass index, n (%)**								
<18.5	86	(18.9)	15	(19.7)	22	(13.8)	49	(22.2)
≥18.5 and <25	304	(66.7)	53	(69.7)	114	(71.7)	137	(62.0)
≥25	66	(14.5)	8	(10.5)	23	(14.5)	35	(15.8)
**Smoking, n (%)**	128	(28.1)	9	(11.8)	37	(23.3)	82	(37.1)
**Alcohol drinking, n (%)**	174	(38.2)	17	(22.4)	67	(42.1)	90	(40.7)
**Parity, n (%)**								
Primiparity	357	(78.3)	67	(88.2)	121	(76.1)	169	(76.5)
Multiparity	99	(21.7)	9	(11.8)	38	(23.9)	52	(23.5)
**Spontaneous abortion, n (%)**	72	(15.8)	18	(23.7)	18	(11.3)	36	(16.3)
**Artificial abortion, n (%)**	100	(21.9)	15	(19.7)	31	(19.5)	54	(24.4)
**Fertility treatment, n (%)**	26	(5.7)	12	(15.8)	12	(7.5)	2	(0.9)
**Planned pregnancy, n (%)**	160	(35.1)	50	(65.8)	55	(34.6)	55	(24.9)
**Diseases under treatment, n (%)**								
Epilepsy	178	(39.0)	45	(59.2)	73	(45.9)	60	(27.1)
Headache*	18	(3.9)	1	(1.3)	10	(6.3)	7	(3.2)
Other neurological diseases^†^	18	(3.9)	5	(6.6)	5	(3.1)	8	(3.6)
Mental and behavioural disorders	273	(59.9)	35	(46.1)	87	(54.7)	151	(68.3)
Collagen diseases	6	(1.3)	0	(0)	2	(1.3)	4	(1.8)
Others	80	(17.5)	16	(21.1)	22	(13.8)	42	(19.0)

*Headache including migraine and cluster headache. ^†^Other neurological diseases except epilepsy and headache.

“Planned pregnancy, n (%) Unplanned pregnancy”

now reads:

“Planned pregnancy, n (%)”

Finally, in the Discussion

“In the present study, a planned pregnancy was the strongest factor associated with folic acid intake”

now reads:

“In the present study, a planned pregnancy was the strongest factor associated with adequate folic acid intake”

